# Cardiovascular Health during and after Cancer Therapy

**DOI:** 10.3390/cancers12123737

**Published:** 2020-12-11

**Authors:** Kathryn J. Ruddy, Shruti R. Patel, Alexandra S. Higgins, Saro H. Armenian, Joerg Herrmann

**Affiliations:** 1 Department of Oncology, Mayo Clinic, Rochester, MN 55905, USA; higgins.alexandra@mayo.edu; 2 Department of Internal Medicine, Mayo Clinic, Rochester, MN 55905, USA; Patel.Shruti@mayo.edu; 3 Department of Population Sciences, City of Hope, Duarte, CA 91010, USA; sarmenian@coh.org; 4 Department of Cardiovascular Disease, Mayo Clinic, Rochester, MN 55905, USA; Herrmann.Joerg@mayo.edu

**Keywords:** cardio-oncology, anthracycline, congestive heart failure, myocarditis

## Abstract

**Simple Summary:**

This review aims to summarize how cancer treatments (both new and old) can impair heart and blood vessel health, and what can be done to prevent or treat these issues. We discuss lifestyle, medication, and other approaches to consider in this setting.

**Abstract:**

Certain cancer treatments have been linked to specific cardiovascular toxicities, including (but not limited to) cardiomyopathy, atrial fibrillation, arterial hypertension, and myocarditis. Radiation, anthracyclines, human epidermal growth factor receptor 2 (Her2)-directed therapies, fluoropyrimidines, platinums, tyrosine kinase inhibitors and proteasome inhibitors, immune checkpoint inhibitors, and chimeric antigen-presenting (CAR)-T cell therapy can all cause cardiovascular side effects. Management of cardiovascular dysfunction that occurs during cancer therapy often requires temporary or permanent cessation of the risk-potentiating anti-neoplastic drug as well as optimization of medical management from a cardiovascular standpoint. Stem cell or bone marrow transplant recipients face unique cardiovascular challenges, as do patients at extremes of age.

## 1. Introduction

Cardiovascular disease (CVD) is a common cause of morbidity and mortality in cancer survivors, in part due to complications of cancer therapy (e.g., myocardial dysfunction, coronary artery or peripheral vascular disease, valvular disease, arrhythmias, arterial hypertension, and thromboembolism). The cardiotoxicity of anthracyclines and trastuzumab has been long-recognized, as has radiation-induced heart disease. Over the past decade, the range of cardiovascular toxicities has expanded due to the introduction and rapid uptake of numerous targeted therapies and immunotherapies. Patients at extremes of age face unique challenges related to these toxicities. This review will provide an overview of current practices related to primary and secondary prevention strategies to improve cardiovascular health during and after cancer therapy, including in relation to toxicities emerging with newer anti-neoplastic agents.

## 2. Cardiovascular Health Risks with Specific Cancer Therapies

A number of anti-neoplastic medications have been shown to increase the risk of specific CVDs, including (but not limited to) cardiomyopathy, atrial fibrillation, and arterial hypertension [1]. Management of cardiovascular dysfunction that occurs during cancer therapy often requires temporary or permanent cessation of the risk-potentiating anti-neoplastic drug as well as optimization of medical therapy for CVD, often including a beta-blocker (BB) and an angiotensin-converting enzyme inhibitor (ACEI) or angiotensin receptor blocker (ARB), and sometimes an anti-arrhythmic medication [2].

### 2.1. Radiation

A wide range of malignancies are effectively treated with ionizing radiation, which can have long-term cardiovascular effects including vascular, valvular, pericardial, and myocardial damage, impairing the long-term health of survivors. Population-based studies have found increased cardiovascular mortality more than 15 years after radiation therapy [3]. Mediastinal or chest radiation can induce fibrosis of small vessels, conducting tissues, and cardiomyocytes, leading to arrhythmias, pericarditis, and effusions. In addition, radiation can increase atherosclerosis, leading to myocardial ischemia that can cause heart failure. This is a contributor to the elevated rates of cardiovascular disease seen in lymphoma survivors [4,5], including pericardial diseases, valvular diseases, and atherosclerotic disease (e.g., worse outcomes after percutaneous coronary intervention in Hodgkin’s Lymphoma survivors compared to the general population) [6]. Preventing radiation-induced heart disease is largely dependent on exposing the cardiac tissue to the lowest effective dose of radiation, as cardiotoxicity increases linearly with radiation dose (with no clear safe threshold) [7]. 3-dimensional conformal RT using focused radiation beams, intensity-modulated RT (IMRT), or proton therapy can minimize the dose of ionizing radiation to cardiac structures adjacent to a tumor [8]; long-term studies are needed to determine how much these modalities decrease risk of late onset cardiac dysfunction. In contrast, radioprotectors to counteract effects of ionizing radiation have been poorly tolerated and infrequently utilized [9].

### 2.2. Anthracyclines

Cardiotoxicity is a dose-limiting side effect of anthracyclines (e.g., doxorubicin, daunorubicin, idarubicin, epirubicin), likely in part due to topoisomerase II inhibition and the generation of free radicals and reactive oxygen species, which can damage cardiac myocytes [10,11]. Importantly, there are two types of cardiotoxicity associated with anthracycline therapy: acute and chronic. The acute form is rare and generally attributed to an inflammatory response to the drug; this potentially fatal reaction typically manifests with pericarditis, arrythmia, and left ventricular dysfunction [12]. Chronic anthracycline cardiotoxicity is more common, and is thought to be caused by apoptotic cardiomyocyte death [11]. Assuming that acute anthracycline-induced cardiotoxicity is not fatal, patients more often experience recovery in EF than with chronic anthracycline-induced cardiotoxicity. Historically, anthracycline-induced chronic cardiomyopathy was associated with a 60% mortality within 2 years [13], but five-year survival rates have been considerably higher (>80%) recently [14]. In cultured cardiomyocytes and rodent models, anthracyclines have a pro-arrhythmic effect, and real-world data indicate that the burden of arrhythmia in patients with anthracycline-related cardiomyopathy is similar to that in patients with ischemic heart disease or dilated cardiomyopathy in patients with and without cancer [15].

Asymptomatic reductions in EF can occur during or soon after completion of anthracycline, but symptoms may not develop until years later. Some affected patients with EF > 40% for whom ongoing anthracycline treatment will provide great benefit (e.g., those with metastatic breast cancer that has been refractory to other agents but is responding to an anthracycline) can continue on anthracycline with close monitoring and pharmacologic management [11].

In part due to concerns related to the deterioration of cardiac function that occurs with higher cumulative doses, anthracycline-based therapies are less popular than they used to be, at least for patients with early-stage breast cancer. However, anthracyclines are still administered to thousands of women with early-stage breast cancer each year in the United States, as well as to many women with metastatic breast cancer (who often end up receiving an anthracycline as one of many sequential lines of treatment for incurable disease, sometimes with high total cumulative doses). Additionally, treatment of many other cancers, especially lymphomas, leukemias, and sarcomas, continue to rely on anthracyclines. Dexrazoxane, an intracellular iron chelator preventing oxygen free radical formation and inhibiting topoisomerase II beta isoenzyme, is the only approved agent for preventing anthracycline-induced cardiotoxicity. Its FDA approval is restricted to use in patients who have experienced anthracycline extravasation into the skin and subcutaneous tissue, as well as in those with metastatic breast cancer who have received a cumulative dose of 300 mg/m^2^ with plans to continue on doxorubicin [16]. Alternatively, liposomal doxorubicin can sometimes be used instead of standard doxorubicin (particularly for those with incurable disease, who will need prolonged treatment) to reduce the risk of cardiotoxicity by approximately 50% [17,18]. Patients who need anthracycline-based therapy but are at high risk of cardiotoxicity may benefit from the consideration of cardioprotective medications such as a beta-blocker (e.g., carvedilol, nebivolol, or bisoprolol), ACE-I/ARBs [19], and/or statin. Some studies have identified improved outcomes with use of ACEI/ARBs in anthracycline recipients [20,21,22], but more research is needed regarding the optimal use of cardio-protective medications in this setting.

### 2.3. HER2-Directed Therapies

Human epidermal growth factor receptor-2 (HER2)-targeted agents are effective against tumor types that overexpress HER2 [23], including 20% of breast cancers, as well as some gastric and colorectal cancers [24]. HER2-targeted agents are well-known to carry a risk of cardiomyopathy that is typically reversible, though the pathophysiology of this toxicity is still unclear. In mouse studies, HER2 seems to be important in cardiovascular development [25], but this does not fully explain the reversible chemotherapy-related cardiac dysfunction seen in humans with trastuzumab, a monoclonal antibody against HER2 [26]. Every-three-month echocardiography is recommended to detect asymptomatic declines in EF during trastuzumab therapy, at least in the early-stage disease setting [27]. Trastuzumab therapy is often held or discontinued when reduced EF is detected, though in the metastatic setting, detection of asymptomatic reductions in EF is less valuable given the critical disease-control benefit of continuing HER2-targeted treatment.

Whether or not trastuzumab therapy can be safely used in patients with a reduced cardiac function at baseline has been assessed in several studies. One retrospective analysis by Nowsheen et al., found that women with reduced cardiac function at baseline had no higher risk of LVEF decline with trastuzumab therapy, although they were greater than five times more likely to experience symptomatic heart failure [28]. In the SAFE-HEART and SCHOLAR prospective pilot trials, 90% of patients with an LVEF in the mid-range (LVEF 40–49%) were able to complete the planned course of trastuzumab therapy [29,30]. The relatively low event rates in these prospective trials support the notion that with optimal treatment and close follow-up in the cardio-oncology clinic, most patients with EF >40% can be safely treated with trastuzumab.

Prevention of trastuzumab-associated cardiotoxicity has been of great interest for patients with early-stage breast cancer. The MANTICORE trial demonstrated a benefit for bisoprolol rather than perindopril in primary prevention of declines in EF during HER2-directed therapy, but there was no significant difference in ventricular remodeling between the arms of the study [31]. In the SCUSF 0806 Trial, both lisinopril and carvedilol appeared to independently prevent cardiotoxicity in patients treated with trastuzumab after prior exposure to anthracyclines. An ongoing randomized trial, the TrAstuzumab Cardiomyopathy Therapeutic Intervention with Carvedilol (TACTIC) Trial (NCT03879629), is assessing the value of biomarker-based compared to universal upfront initiation of carvedilol in this setting. The three arms of TACTIC, which will be compared with regard to protection against EF declines and congestive heart failure, include: (1) carvedilol prior to HER2-directed therapy; (2) carvedilol initiated after documentation of subclinical cardiac dysfunction/injury, defined by an abnormal global longitudinal strain (GLS) or cardiac troponin (cTn) elevation; and (3) carvedilol initiated after onset of congestive heart failure or a drop in EF (by >10% in patients whose LVEF is ≥50%, or by ≥5% in those with a decrease to< 50%). Other studies have demonstrated that GLS can serve as a subclinical marker of left ventricular dysfunction during or after receipt of a cardiotoxic medication [32,33]. TACTIC will also evaluate if carvedilol continuation for an additional year after completion of HER2-directed therapy provides more effective cardioprotection than cessation of carvedilol at the time of completion of HER2-directed therapy.

Most new HER2-directed agents (e.g., lapatinib, ado-trastuzumab emtansine, pertuzumab, fam-trastuzumab derutxtecan, and tucatinib) seem to carry less risk of cardiotoxicity than trastuzumab [34,35,36,37,38], though confirmation of a normal EF is still currently recommended prior to starting treatment and periodically during treatment with many HER2-targeting drugs including lapatinib, pertuzumab, and fam-derutxtecan [37,39,40,41]. A meta-analysis concluded that adding lapatinib or pertuzumab to traztuzumab therapy does not increase cardiotoxicity [42]. A pooled analysis of ado-trastuzumab emtansine trials demonstrated low incidence of cardiac events at 3.37% including ischemia, CHF, and arrhythmia [35]. The most recently FDA-approved agent, fam-trastuzumab derutxtecan, has the lowest rate of asymptomatic LVEF decrease at 0.9% of patients. This medication has yet to be studied in patients with known cardiac disease or LVEF < 50% [37].

### 2.4. Cyclophosphamide

It is rare for patients to develop cardiovascular compromise related to cyclophosphamide without concomitant use of an anthracycline. When cyclophosphamide cardiotoxicity does occur, it seems to usually manifest 1–3 weeks after receipt of a single high-dose infusion of the drug (such as for a stem cell transplant) rather than after receipt of multiple doses that sum to a cumulatively high dose. Cyclophosphamide cardiotoxicity may be related to endocardial injury followed by extravasation of toxic metabolites that can damage cardiac myocytes. Arrhythmias, congestive heart failure, myopericarditis, pericardial effusion, and death can result [43,44].

### 2.5. Fluoropyrimidines

Fluoropyrimidine-based therapies (e.g., 5-FU and capecitabine), are commonly used agents in the treatment of solid tumor malignancies. 5-FU cardiotoxicity affects 1.2 to 18% of treated patients [45,46]. The most common clinical manifestation of this cardiotoxicity is chest pain, typical or atypical [47,48]. MI, arrhythmia, myocarditis/pericarditis, and heart failure are also possible [49]. Vasoconstriction of the epicardial vasculature and even the microcirculation has been considered to play an important pathomechanistic role. Vasodilatory agents, however, have not been consistently shown to prevent 5-FU cardiotoxicity, suggesting that there are alternative mechanisms at play and possibly a more important role of the coronary microcirculation and resistance to vasodilatory efforts (Takotsubo-like presentation) [46]. Capecitabine, an oral pro-drug of 5-FU, can lead to similar presentations with some studies suggesting lower and others comparable cardiotoxicity rates to 5-FU [50].

Cardiotoxicity from fluoropyrimidine-based therapy is largely reversible, even if a decline in cardiac function is seen, unless irreversible injury (i.e., myocardial infarction) has occurred due to prolonged periods of ischemia. Profound and prolonged vasoconstriction can cause ventricular tachycardia and ventricular fibrillation [46]. Due to these risks, in patients with symptoms suggestive of cardiotoxicity, fluoropyrimidines should be immediately discontinued, and anti-anginal treatment (e.g., calcium channel blocker and nitrate) should be initiated [51]. There are variable data on the safety of re-challenging these patients such that this practice is controversial [51,52]. If 5-FU therapy cannot be avoided, a bolus regimen with 48 h pretreatment with both a calcium channel blocker and a long-acting nitrate, with continuous ECG monitoring, can be considered (with 5-FU discontinuation if any sign or symptom of another cardiac event develops) [49]. Uridine triacetate has been approved for the use in patients with severe/life-threatening 5-FU toxicity, including those of cardiovascular nature.

### 2.6. Platinums

Platinums (e.g., cisplatin, carboplatin, and oxaliplatin) are used to treat a variety of solid organ tumors including testicular, ovarian, cervical, bladder, lung, and head and neck [53]. Long-term effects of platinums have been most well-studied in patients with testicular cancer, with vascular toxicity clearly evident in some men [54]. As platinums can be measurable in plasma several years after therapy [55], it is theorized that these agents could continuously act on the endothelium over years, causing endothelial dysfunction [1]. The consequences of this, including early atherosclerosis, coronary artery disease, and thromboembolic events, may be exacerbated by the adverse metabolic risk factor profile (e.g., diabetes, obesity) that is present in some of these patients [56]. Monitoring for signs and symptoms of vascular disease during long-term follow-up is warranted in prior recipients of platinums.

### 2.7. Tyrosine Kinase Inhibitors and Proteasome Inhibitors

Tyrosine kinase inhibitors (TKIs, e.g., sunitinib, sorafenib, pazopanib, axitinib, vandetanib, cabozantinib, ponatinib and regorafenib) and monoclonal antibodies (e.g., bevacizumab) that inhibit the vascular endothelial growth factor receptor are used to treat a wide variety of malignancies. These agents carry a risk of arterial hypertension, symptomatic or asymptomatic heart failure, and prolonged QT [57]. Importantly, the incidence of cardiovascular toxicity differs considerably between TKIs, with multiple pathways involved in TKI-cardiotoxicity [58,59,60]. Proteasome inhibitors (e.g., bortezomib and carfilzomib, but especially carfilzomib), used to treat multiple myeloma and related conditions, are also known to increase risk of myocardial dysfunction [61].

Osimertinib, a third generation TKI, recently approved for first line therapy of advanced non-small cell lung cancer, has been reported to cause adverse cardiac events including acute myocardial infarction (MI), heart failure with reduced LVEF, and valvular heart disease in approximately 4–5% of patients [62,63,64]. A recent review of reported cases illustrates the benefit of cardioprotective medications such as BB and ACEI for improving EF in this setting and suggests that this cardiomyopathy is reversible [65].

Ibrutinib, a Bruton’s tyrosine kinase (BTK) inhibitor, effectively treats various hematologic malignancies including many B-cell lymphomas, but atrial fibrillation can occur in 4–16% of patients on ibrutinib [66]. Because cancer causes hypercoagulability, atrial fibrillation is associated with a higher risk of thromboembolism in these patients than in the general population. Interestingly, ibrutinib also impairs platelet activation, increasing the risk of bleeding on anticoagulation. The management of ibrutinib-associated CVD is further complicated by many drug-drug interactions (including with calcium channel blockers, digoxin, amiodarone, and direct oral anticoagulants) [67].

### 2.8. Immune Checkpoint Inhibitors

With the widespread use of immune checkpoint inhibitors (ICIs) across oncology, immune related adverse events (irAEs) have gained noteworthy recognition. Myocarditis is one of the least common (occurring in only 0.6–1% of people treated with ICIs), but one of the most serious irAEs with a reported mortality rate in the range of 40–60% [68]. Myocarditis is diagnosed if any of the following criteria are met: (1) tissue pathology demonstrates myocarditis; (2) cardiac MRI is diagnostic of myocarditis with either elevated biomarkers or ECG evidence of myo-pericarditis; or (3) there are new wall motion abnormalities on echocardiogram that cannot be explained by another diagnosis plus a clinical syndrome consistent with myocarditis, elevated cardiac biomarkers, ECG evidence of myo-pericarditis, and negative evaluation for coronary artery disease [69,70,71]. Myositis and myasthenia gravis occur concurrently in more than one third of patients with severe ICI-induced myocarditis. Risk of ICI-associated myocarditis is higher when anti-CTLA-4 therapy is combined with anti-PD-L1 therapy than with anti-PD-L1 monotherapy [72], and risk may be elevated for patients with autoimmune disease and diabetes mellitus. Myocarditis typically presents with an elevated troponin and ECG changes (commonly ventricular ectopy, block patterns, ST segment changes) while cardiac imaging findings can be strikingly absent or subtle (e.g., reflected only in an abnormal GLS) [73]. Higher cardiac troponin and abnormal GLS may indicate a more complicated disease course. Time from initiation of ICI to diagnosis is often short; median time was 34 days in a 35-case multicenter series [74]. The risk of developing a major adverse cardiac event (defined as the composite of cardiovascular death, cardiogenic shock, cardiac arrest, and hemodynamically significant complete heart block) is nearly 50% with this condition [74]. Symptoms are often very similar to those of acute coronary syndrome and acute cardiomyopathy from other etiologies, potentially delaying treatment, which should be high-dose steroids upon clinical suspicion (methylprednisolone 1 g/day for 3–5 days) [72]. While outcomes are worse with delays and undertreatment, patients can experience a rapid clinical deterioration even with the administration of high dose steroids; in that case, escalation of immunosuppressive therapy is recommended, and plasmapheresis is considered. Patients with fulminant myocarditis progressing to cardiogenic shock may require mechanical support, including extracorporal circulatory membrane oxygenation (ECMO).

Cardiology consult, ECG, echocardiogram, and troponin are recommended when myocarditis is suspected. Cardiac MRI with contrast is the imaging modality of choice. An endomyocardial biopsy should be considered if the diagnosis is uncertain, particularly for patients with severe symptoms and no options for additional imaging studies. A T-cell-predominant lymphocytic infiltrate is commonly seen on biopsy, although this can be missed due to a patchy distribution. Steroids should be continued until cardiac function normalizes. A slow (4–6 week) steroid taper is recommended by the 2020 National Comprehensive Cancer Network (NCCN) Management of Immunotherapy-Related Toxicities Guideline Panel [72]. Those patients with life-threatening events on ICI should not be resumed on ICI therapy.

### 2.9. CAR-T Cell Therapy

Chimeric antigen-presenting T (CAR-T) cell therapy, an exciting advance in hematologic oncology, can impact the cardiovascular system. In a recently published cohort of 137 patients who received CAR-T, 17/137 (12%) experienced a cardiac adverse event (including 6 cardiovascular deaths, 6 decompensated heart failure events, and 5 arrhythmias). Median time from CAR-T administration to cardiac event was 21 days. Twenty-nine of 53 (54%) patients who underwent troponin assessment were found to have elevated levels, and 8/29 (28%) of those who underwent echocardiography were found to have a decreased left ventricular EF [75]. There are limited data on the long-term complications associated with CAR-T cell therapy, but most cardiovascular complications appear to be transient and occur early [76].

### 2.10. Special Considerations before, during, and after Stem Cell or Bone Marrow Transplants

Hematopoietic cell transplantation (HCT) is an established and effective treatment for many hematological disorders and malignancies [77]. Improvement in HCT strategies during the past five decades has led to an increasing number of long-term survivors [78,79], but their mortality rates remain substantially higher than those in the general population [80,81,82]. In fact, the risk of cardiovascular-related mortality after HCT is more than twice that of the general population [81,82,83], and the magnitude of risk increases with time from HCT [83,84,85]. In HCT survivors, cardiovascular complications such as MI, stroke, and heart failure can cause long-term morbidity [86]. Outcomes following onset of CVD in HCT survivors are poor; five-year survival is less than 50% [85,87], emphasizing the need for innovative and risk-based prevention strategies that address the changing burden of CVD over time after HCT.

To date, there have been limited evidence-based interventions to reduce CVD risk in HCT survivors, though there are well-described pre-HCT (e.g., anthracycline chemotherapy, chest radiation), conditioning-related (e.g., high-dose cyclophosphamide), and post-HCT (e.g., modifiable cardiovascular comorbidity [hypertension, diabetes, dyslipidemia]) risk factors for these health conditions [83,84,85]. A recent combined effort using data from two large HCT survivorship cohorts produced a validated model to stratify HCT survivors into low-, intermediate-, and high-cardiovascular risk groups, corresponding to 10-year cumulative incidences of CVD of 3.7%, 9.9%, and 26.2%, respectively [88]. In this model, hypertension, diabetes, and obesity are all important modifiable risk factors for CVD [88]. This model’s post-HCT start time point capitalizes on the “teachable moment”, meaning that patients who have recently survived one life-threatening disease may be motivated to try to prevent additional illness. However, patients are at risk of developing modifiable risk factors for CVD over many years afterwards, during the period when there is often a decline in engagement with cancer-centered care [85,86]. New paradigms of survivorship care are needed to address the well-documented high rates of underdiagnosis and undertreatment of these CVD risk factors in HCT survivors [89,90].

## 3. Primary and Secondary Prevention after Cancer Therapy

### 3.1. ABCDE

**Figure 1:**
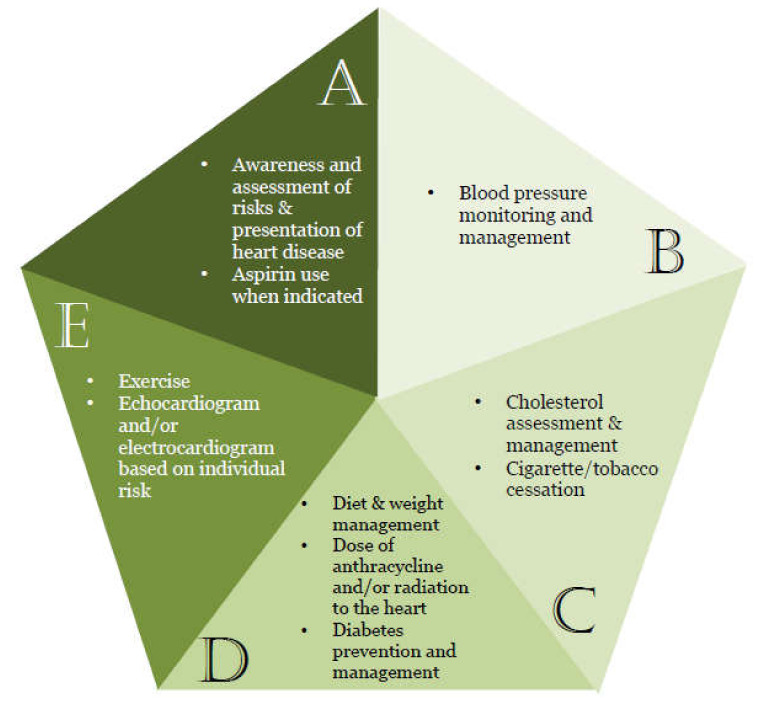
ABCDEs to promote Cardiovascular Health in Cancer Survivors.

Primary and secondary prevention of CVD is important in all cancer survivors, but especially critical in those exposed to cardiotoxic therapies. Key elements in cardio-oncology care for survivors can be captured in the ABCDE acronym, (Figure 1) [91]. A key first step is awareness of the long-term cardiovascular risks in cancer survivors and the need for their assessment/monitoring. These are particularly important after the use of higher doses of anthracyclines (>250 mg/m^2^) and chest radiation therapy (>30 Gy), though any exposure may generate risk. The most common surveillance strategy is currently cardiac function assessment by echocardiography. The other recommendations for survivors are concordant with the current primary prevention guidelines [92]. These include the ASCVD risk score for general CV risk assessment [93]. While aspirin use is indicated for secondary prevention, only high risk individuals meet criteria for aspirin in the primary prevention setting. The goal for treating blood pressure should be 130/80 mmHg, and therapy is to be initiated if this value is exceeded in the presence of an ASCVD 10-year risk >10%, or if BP is >140/90 mmHg. High intensity statin is recommended for those with low density lipoprotein (LDL) cholesterol 190 mg/dL or higher or ASCVD risk >20% (with the treatment goal being a ≥50% LDL reduction). A moderate intensity statin (with a 30–49% LDL reduction treatment goal) is recommended for those with diabetes or an ASCVD risk 7.5% to <20%. A diet emphasizing vegetables, fruits, legumes, nuts, whole grains, and fish is also recommended. All cancer survivors should perform at least 150 min per week of moderate-intensity physical activity or 75 min of vigorous-intensity physical activity; this is especially important for those with diabetes (to improve glycemic control) and obesity.

For survivors who have already experienced cardiovascular events, secondary prevention measures should be in place [94]. The AHA recently proposed the development of a cardiac rehabilitation program for cancer patients and survivors with a focus on structured exercise with trained professionals managing the ABCDEs of cardio-oncology care [95].

### 3.2. Monitoring Cardiovascular Health in Survivors

A survivor’s individualized cardiovascular risks after completion of cancer therapy can help guide surveillance decisions. Risk assessment is dependent on drugs and doses received, whether or not radiation therapy was given to a field that included the heart, and other cardiac risk factors/history. Some studies have identified sex-based differences in cardiac outcomes in cancer survivors [96], but additional research is needed to clarify how these might impact surveillance. Based on the American Society of Clinical Oncology (ASCO) expert consensus, patients who have received anthracyclines are considered to be at high risk for poor cardiovascular health if they possess multiple cardiac risk factors (e.g., smoking, hypertension, diabetes, dyslipidemia, and obesity), were treated with high-dose anthracycline (e.g., ≥250 mg/m^2^ doxorubicin or ≥600 mg/m^2^ epirubicin), or if they were also treated with radiotherapy that included the heart in the treatment field or trastuzumab [97]. Patients who have received trastuzumab without anthracycline are only considered to be high risk if they have multiple cardiac comorbidities, age >60 years old, or compromised cardiac function. The optimal approach to monitoring cardiovascular health in survivors is controversial. In pediatric survivors, the Children’s Oncology Group recommends echocardiography at least every five years for all anthracycline recipients (and more often for those treated with high doses and/or radiation) [72]. In adult survivors, ASCO and NCCN recommend consideration of a single echocardiogram 6–12 months after completion of anthracycline (with no later assessments if that echocardiogram is normal) [97,98]. However, our recent evaluation of administrative claims data from OptumLabs identified low rates of post-anthracycline echocardiography, even in patients over age 65 [99]. European Society of Cardiology (ESC) recommends use of brain natriuretic peptide (BNP) as a serum-based biomarker to identify patients with Stage A heart failure (which includes all anthracycline recipients) who could benefit from further cardiac investigation such as echocardiography [100].

## 4. Cardiovascular Considerations at Extremes of Age

**Figure 2:**
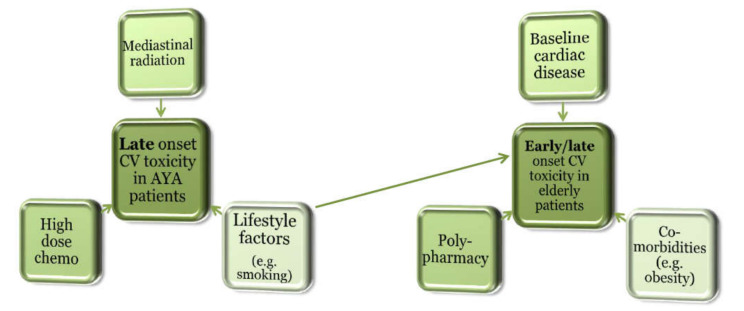
Common contributors to cardiovascular disease after cancer at extremes of age.

Cardiovascular sequelae of treatment are common in adolescent and/or young adult (AYA) patients with Hodgkin’s lymphoma, non-Hodgkin’s lymphoma, Wilms tumors, breast cancer, and mediastinal testicular cancers, which are often treated with mediastinal radiation [8,101]. Some of these also require anthracycline therapy, compounding risks. Consequently, survivors of Hodgkin’s lymphoma treatment have two to eight times higher risk of fatal MI than the general population; median time to this diagnosis is approximately two decades after cancer treatment [102,103,104]. A study of over 5000 survivors of AYA cancer found a two-fold increased risk of developing CVD compared to the general population, and cancer survivors who developed CVD had an 11-fold increase in overall mortality risk compared to survivors without CVD (Figure 2) [105].

Further research efforts are underway to identify effective and tolerable cardioprotective strategies for survivors of AYA cancers. The Children’s Oncology Group recommends screening echocardiography (with the frequency dependent on cumulative risk and co-morbidities), assessment of comorbid conditions that may affect the risk of CVD (e.g., excess weight, hypertension, diabetes mellitus, and dyslipidemia), and heart-healthy lifestyle counseling [8] in this population.

Care of the elderly patient with cancer is also a unique challenge for oncologists as most patients will have comorbid conditions, many of which increase the risk of cardiovascular toxicity. Common co-morbidities in patients over 60 years of age include hypertension, hyperlipidemia, heart failure, diabetes, and ischemic heart disease with prevalence of each ranging from 20 to 75% [106]. Most observational studies have identified higher mortality rates among the elderly with increasing number of co-morbidities [107]. American Society of Clinical Oncology guidelines state that risk of cardiac dysfunction increases with age and estimate that cardiotoxicity is 1.6 to 6.8-fold higher in elderly patients compared to young patients [97]. Despite the increased risk of treatment related toxicities, there has also been concern that cancer in the elderly is sometimes undertreated due to overestimated risks of treatment [108,109], and further evidence-based guidance is needed to properly risk stratify elderly patients with cancer with regard to cardiovascular risks. Investigations into potential biomarkers for cardiotoxicity are underway, including myocardial strain, cardiac troponin, and brain natriuretic peptide [110].

## 5. Next Steps

The burgeoning interest in telemedicine and remote patient monitoring may provide improved methods for improving the management of health conditions, including CVD in patients with cancer [111,112,113]. Related technologies preceded but are now accelerated by the recent COVID-19 pandemic [114]. Studies have demonstrated the feasibility of implementing remote cancer survivorship care delivery in high-risk patients [115,116,117], setting the stage for the development of population-based CVD interventions in large cohorts of survivors at a fraction of the cost that in-person care requires. These efforts set the stage for innovative delivery of care that will encourage patients to be actively engaged in CVD prevention and treatment during and after cancer therapy.
